# Comparing reproductive intentions before and during the COVID-19 pandemic: a cross-sectional study

**DOI:** 10.1186/s12913-023-09551-z

**Published:** 2023-05-25

**Authors:** Fatemeh Rezaei, Leila Amiri-Farahani, Shima Haghani, Sally Pezaro, Fereshteh Behmanesh

**Affiliations:** 1 Department of Reproductive Health and Midwifery, School of Nursing and Midwifery, Iran University of Medical Sciences, Tehran, Iran; 2 Department of Reproductive Health and Midwifery, Nursing and Midwifery Care Research Center, School of Nursing and Midwifery, University of Medical Sciences, Tehran, Iran; 3 Department of Biostatistics, Nursing and Midwifery Care Research Center, Iran University of Medical Sciences, Tehran, Iran; 4 The Research Centre for Healthcare and Communities, Coventry University, Coventry, UK; 5 The University of Notre Dame, Notre Dame, Australia; 6 Social Determinants of Health Research Center, Health Research Institute, Babol University of Medical Sciences, Babol, I.R. of Iran

**Keywords:** Reproductive behaviors, COVID-19 pandemic, Reproductive intentions

## Abstract

**Background and Aim:**

Reproductive behaviors and intentions are affected by several factors, including the COVID-19 pandemic crisis. This study was conducted with the aim of comparing the intention to reproduce and its causes in Iran during the period prior to and during the COVID-19 pandemic.

**Methods:**

This descriptive-comparative study included 425 cisgender women from 6 urban health centers and 10 rural centers in Babol city, Mazandaran province, Iran. Urban and rural health centers were selected using a multi-stage approach with proportional allocation. A questionnaire was used to collect data in relation to individual characteristics and reproductive intentions.

**Results:**

Most participants aged between 20 and 29 years had a diploma level of education, were housewives and lived in the city. The intention to reproduce decreased from 11.4% before the pandemic to 5.4% during the pandemic (p = 0.006). The most common reason for wanting to have children prior to the pandemic was not having children (54.2%). During the pandemic, a desire to reach the ideal number of children was the most common reason to want to have children (59.1%), though no statistically significant difference was observed between the two periods (p = 0.303). The most common reason for not wanting to have children in both periods was having enough children already (45.2% before and 40.9% during the pandemic). The reasons for not wanting to have children had a statistically significant difference between the two periods (p < 0.001). Reproductive intentions had a statistically significant relationship with the variables of age (p < 0.001), the education level of both participants (p < 0.001) and spouses (p = 0.006), occupation (p = 0.004), and socio-economic status (p < 0.001).

**Conclusion:**

Despite restrictions and lockdowns, the COVID-19 pandemic had a negative impact on people’s desire to reproduce in this context. Economic problems caused by the sanctions which increased during the COVID-19 crisis may be one of the reasons for a reduction in people’s intentions to become parents. Future research could usefully investigate whether this decrease in the desire to reproduce will lead to significant changes in population level and future birth rates.

**Supplementary Information:**

The online version contains supplementary material available at 10.1186/s12913-023-09551-z.

## Introduction

Reproductive intention is the expression of fertility desire based on an individual or family preference for children, taking into account various limitations such as the ideal number of children, gender/sex, time, and interval between pregnancies [1]. Reproductive intentions are influenced by many factors including the economy, policies, levels of education, the environment, and public services [2]. Various factors such as age, family income, ethnicity, education [3, 4], religion [5, 6], gender preference [6], age of marriage, satisfaction with married life [7, 8], number of living children [3, 8], employment [3], length of marriage, attitudes about the use of contraceptives [7] and place of residence [5] influence reproductive behaviors and the desire to have children. Other factors affecting reproductive behaviors are crises caused by natural disasters such as floods, earthquakes, and storms. These crises cause reduced access to contraceptive services and can also lead to short-term reductions in reproduction [9, 10]. Thus, it is useful to consider what impacts such crises may have on reproductive behaviors.

The crisis of the COVID-19 pandemic presented the world with many sexual and reproductive health challenges in society [11]. The COVID-19 pandemic has also led to increased unemployment and loss of health insurance for many people, resulting in reduced access to health care [12]. Social distancing and disruptions caused by these pandemic created physical and economic barriers for pregnancy prevention and sexual and reproductive health services. Moreover, it has affected individuals’ reproductive priorities and feelings towards having children [13, 14]. Quarantine and the presence of more family members at home may have also affected individuals’ sexual activity [15]. Nevertheless, it is as yet unclear as to how these effects may have wholly manifested themselves.

A survey by Lindberg colleagues (2020) in the United States of America (USA) showed changes in the desire to have children in 40% of their participants, and these changes were more pronounced in those childless. In addition, 34% reported that they delayed their parenthood plans due to the COVID-19 pandemic, and 17% planned to have children due to the COVID-19 pandemic [14]. In Italy, the intention to reproduce during the COVID-19 pandemic was found to be less than before the pandemic, yet 37.3% who had the intention of reproducing before the pandemic were found to have subsequently given up their intention [16]. The main reasons given for this included concerns about future economic problems and the consequences of reproducing. Just 11.5% of individuals who did not intend to have children before the pandemic wanted to have children during quarantine, citing the decision to make a change in life and the need to be positive during this time [16]. The intention to reproduce was been found to be higher in those aged 36–40 prior to the COVID-19 pandemic, and the greatest change in peoples intention to reproduce was reported by those aged between 26 and 30 [16]. A decline in the numbers of pregnancies recorded during the pandemic have been reported more frequently in groups with low-income than in those with high-income, along with black and Hispanic people living in the USA [14]. Further studies in this area may offer more insight into how these changes may have manifested themselves worldwide following COVID-19.

In comparison, Zimmerman et al.‘s (2022) study conducted in Kenya found that the majority of cisgender women (85%) reported stable fertility outcomes both in terms of number and time. No COVID-19-related factors were associated with changes in fertility (Zimmerman et al., 2022). Emery et al.‘s (2022) study conducted in the Republic of Moldova showed that the number of people trying to get pregnant decreased from 8.7% in the pre-pandemic period to 5.7% during the COVID-19 pandemic. However, no difference was identified in the mean duration of fertility between before and after quarantine [17] Limited studies conducted in other countries indicate that the COVID-19 pandemic has resulted in a reduction in the desire to reproduce [14, 16, 18, 19]. Such variations in results around the world demonstrate further need for research in this area.

Those of reproductive age in Babol city represent 22.10% of the total population, and the population of those married and of reproductive age in Iran as a whole is 21.23% of the total population. Prior to the pandemic, the overall birth rate in Babol city was 1.2 per parturient, which is lower than the overall birth rate in Iran (1.8 per parturient) [20]. COVID-19 may have led to changes in this regard, as they have done elsewhere. As such, the present study was designed to compare the desire to reproduce and its causes, and lack of desire to reproduce in Iranian cisgender women before and during the COVID-19 pandemic.

## Methods

### Study design

A cross-sectional study including cisgender women of reproductive age registered with urban and rural health centers in Babol city in Iran was conducted (Supplementary file 1). Informed consent was obtained from participants who had access to WhatsApp software, after providing information about the purpose of the research and the study method and assuring participants’ confidentiality.

### Study sample

To determine the minimum required sample size [21], at the confidence level of 95% and with the accuracy of estimation d = 0.043, after quantification in the following formula, the minimum required sample size was estimated 415.

$$ n=\frac{{\varvec{z}}_{1-\raisebox{1ex}{$\propto $}\!\left/ \!\raisebox{-1ex}{$2$}\right.}^{2} \varvec{p}\varvec{q}}{{\varvec{d}}^{2}}=\frac{{1.96}^{2}\times 0.76\times 0.24}{{0.043}^{2}}=412\approx 415$$

A multi-stage approach was taken with regard to sampling, using the electronic files of the Sib records system of the health centers of Babol city, Mazanaran province, Iran to undertake proportional allocation. The Sib system (integrated health system) hosts the electronic health records of Iranians, and is divided into 5 sections: maternal program (including pregnancy care, pre-pregnancy care, post-natal care, etc.); healthy fertility program (including healthy fertility counseling and childbearing, contraceptive methods, etc.); the middle-aged program; the elderly program, and the children’s program.

Primarily, each of the six districts of Babol city was identified as a stratum. Subsequently, 2–3 rural health centers and 1–3 urban health centers from each stratum were selected at random. Afterward, all electronic codes identified to be a multiple of 15 were selected from the system both systematically and randomly. If selections met our inclusion criteria, those assigned to the record were invited to participate via a telephone call. Sampling from each center occurred from August to November 2021 using the proportional allocation method and continued until a sample size of 425 was reached.

The study inclusion criteria included cisgender women aged between 15 and 49, who had been married for at least 2 years in order to capture marriages prior to the COVID-19 pandemic with subsequent reproductive opportunities [22]. Participants also met the inclusion criteria if they were living with a spouse in the same place, had an active electronic file in the Sib system and willingness join the study. Participants met the exclusion criteria if they reported when asked, primary and secondary infertility, diseases that interfere with an individual’s fertility, or severe mental disorders. In total, 802 electronic files were reviewed, of which 377 did not meet the inclusion criteria.

### Outcome measures and measurements

The data collection tool included a personal profile and a reproductive behavior questionnaire. The personal profile questionnaire was used to collect data in relation to the following: age, level of education, spouse’s education, occupation, and spouse’s occupation, place of residence, socioeconomic status, and weekly rate of sexual intercourse. The validity and reliability of it were confirmed in the current study. The desire to reproduce and the reasons for it and reasons for unwillingness to reproduce were also investigated using the reproductive behavior questionnaire. This questionnaire was designed by Behmanesh et al. in 2015, after which time its validity and reliability were confirmed [23]. This questionnaire contains 20 questions and measures items such as the total number of pregnancies, and information about current pregnancy, abortion, fertility intentions, unwanted pregnancy, causes of unwanted pregnancy, length of time between marriage and first pregnancy, number of children, neonatal mortality and the length of time between each child being born. Questions are asked separately from each other and the only answer to the question “intention to conceive” is a 4-point Likert scale (1. Yes, I currently intend to get pregnant, 2. Yes, I want to get pregnant later, 3. No, I do not intend to get pregnant at all, 4. I don’t know).

In the current study 3 questions relating to reproductive intention, the reasons for the desire to get pregnant and the place where contraceptive methods were obtained were added to the questionnaire. This was then sent to 9 experts, who both quantitatively and qualitatively examined and confirmed content and form validity. As the questions were completely independent from each other, most of the questions were not considered to need checking for reliability. Yet in order to check the reliability of the question “tendency to reproduce” with a Likert scale, Spearman’s correlation was calculated and r = 1 was reported.

Questionnaires were completed using the electronic file information hosted within the Sib system one year prior to the COVID-19 pandemic in Iran (January 2019 to January 2020) and once again during the COVID-19 pandemic (January, 2020 to November 2021) by the researcher (F.R.). Data relating to the number of times sexual intercourse occurred per week, the reasons for wanting to reproduce and the reasons for giving up the desire to reproduce in the period before and during the COVID-19 pandemic were collected via phone call by the researcher (F.R.), as these could not be answered using systematic information.

### Data analysis

Data were analyzed using SPSS software version 26. Descriptive statistics were used to analyze individual characteristics, the desire to reproduce, the causes of the desire and the causes of the unwillingness to reproduce (including absolute and relative frequency, mean and standard deviation). To compare the desire to reproduce, the causes of this desire and the causes of the unwillingness to reproduce, as well as correlations between individual characteristics with the desire to reproduce, chi-square tests were used. The level of significance in all tests was considered p < 0.05.

## Results

### Participants

In total, the electronic files of 802 potential participants aged between 15 and 49 were reviewed, though many files (n = 377) were excluded as they did not meet our inclusion criteria. Reasons for exclusion included: not answering phone calls (n = 237), being dissatisfied with the requirements of the study (n = 48), not living with the spouse in the same place (n = 27), cases of infertility (n = 16), cases of diseases contrary to fertility (n = 2), and cases where the marriage was less than 2 years old (n = 47). Participants were most frequently aged between 20 and 29 years (36.5%) and had a diploma level of education (41.4%). During the COVID-19 pandemic, 79.1% reported to be housewives. Among spouses, the majorities had a university level of education (34.4%), a level of education below a diploma (34.8%), and were self-employed (80.7%). Over half of participants lived in the city (60%), and had relatively favorable socio-economic status (67.1%). Below half had sexual intercourse 1–2 times a week (37%) (Table 1).

**Table 1:** Comparison of reproductive intentions and the reasons for them before and during COVID- 19 pandemic

Reproductive intention		Before the COVID-19 pandemic		During the COVID-19 pandemic		p-Value
		n	%	n	%	
Reproductive intention**	Yes, I want to get pregnant right now	48	11.4	22	5.4	*p < 0.006
	I don’t want to get pregnant right now	158	37.6	150	37.1	
	I do not want to get pregnant at all	215	51.0	232	57.5	
**Reasons for intending to reproduce**	Not having children	26	54.2	9	40.9	*p = 0.303
	To achieve the ideal number of children	22	45.8	13	59.1	
**Reasons for** **not intending to reproduce**	worries about economic difficulties	61	16.4	67	17.5	*p < 0.001
	Disease	11	2.9	10	2.6	
	wrong time	81	21.7	37	9.7	
	Increasing age	15	4.0	11	2.9	
	Spacing of children	54	14.5	75	19.6	
	Sufficient number of children achieved	151	40.5	161	42.2	
	Fear of COVID-19	0	0	21	5.5	

*Chi-square test, Significance level: p < 0.05, bold entries are significant results and are related to statistical test

**Reproductive intention was not asked of 4 pregnant participants

### Reproductive desire

**Fig. 1:**
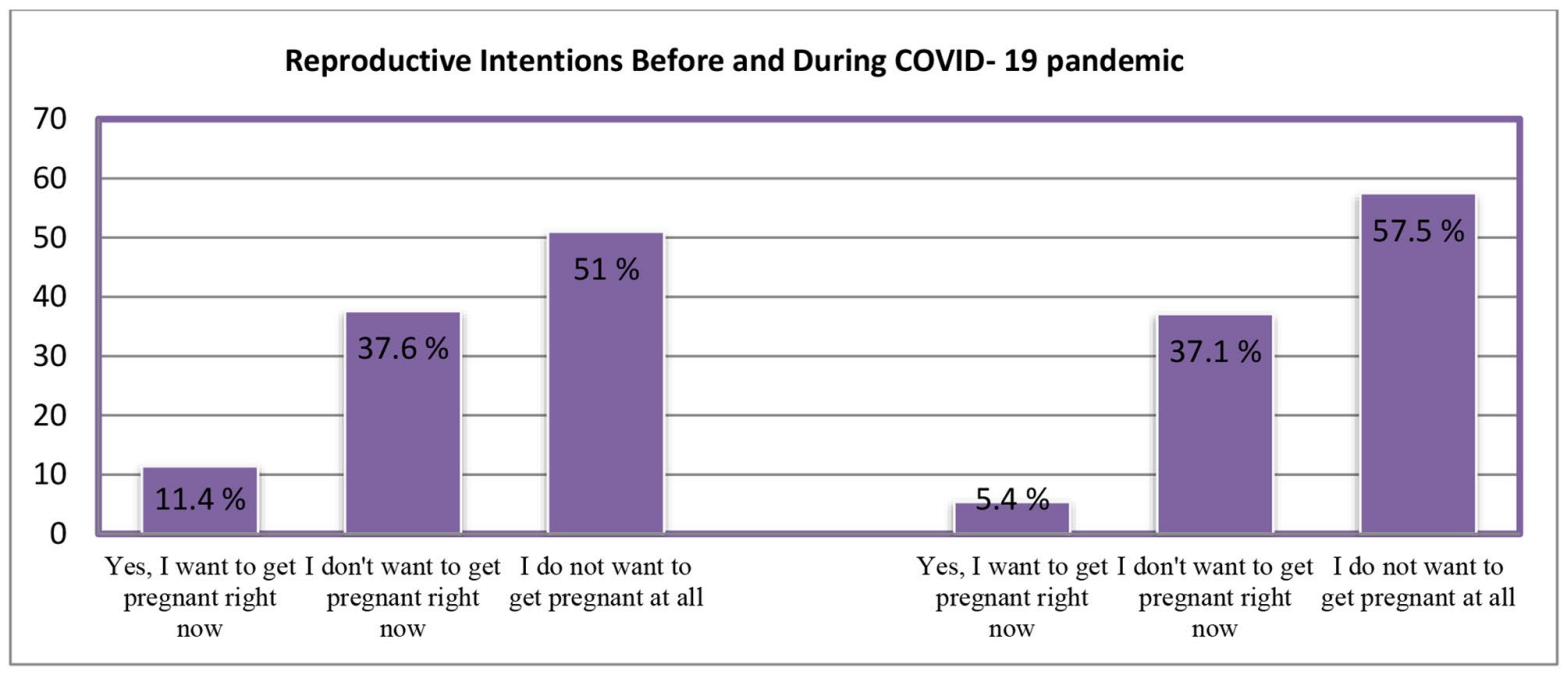
Reproductive intention before and during the COVID-19 pandemic

In the pre-pandemic period, 51% of participants did not want to have children at all. Not having children was the most common reason for wanting to reproduce (54.2%) and having enough children already was the most common reason for not wanting to reproduce in this period (40.5%). Comparisons of participants’ desire to reproduce in the period before and during the COVID-19 pandemic with chi-square test demonstrated a statistically significant difference between the two periods (p = 0.006). Desire decreased from 11.4% in the period prior to the COVID-19 pandemic to 5.4% during the COVID-19 pandemic. Moreover, a statistically significant difference was seen in the reasons for not wanting to reproduce in the previous period compared to during the COVID-19 pandemic (p < 0.001) (Fig. 1; Table 2).

**Table 2:** The relationship between individuals’ characteristics and the reproductive intention during COVID- 19 pandemic

Individual characteristics		Reproductive intention						p-Value
		Yes, I want to get pregnant right now		I don’t want to get pregnant right now		I do not want to get pregnant at all		
		n	%	n	%	n	%	
**Age (years)**	< 20	2	9.5	19	90.5	0	0	0.001 *p <
	20–29	9	6.3	92	64.3	42	29.4	
	30–39	7	4.9	38	26.6	98	68.5	
	40–49	4	4.1	1	1.0	92	94.8	
**Level of education**	< Diploma	1	0.9	30	27.2	79	71.9	0.001 *p <
	Diploma	9	5.4	66	39.7	91	54.9	
	University education	12	9.3	54	42.1	62	48.6	
**Spouses’ level of education**	< Diploma	5	3.5	38	27	98	69.5	0.006 *p =
	Diploma	6	3.2	53	28.6	126	68.2	
	University education	11	8.0	59	43.0	67	49.0	
**Occupation**	Housewife	13	4.1	129	40.6	176	55.3	0.004 *p =
	Employed	9	10.5	21	24.4	56	65.1	
**Spouses’ occupation**	Employee	3	3.8	31	39.7	44	54.5	0.72= *p
	Selfemployed	19	5.8	119	36.5	188	57.7	
**Residence**	Urban	14	5.8	82	33.9	146	60.3	0.25 = *p
	Rural	8	4.9	68	42	86	53.1	
**Socio-economic status**	Unfavorable	3	4.2	23	32.9	44	62.9	0.001 *p <
	Relatively favorable	8	3.0	107	39.8	154	57.2	
	Favorable	11	16.9	20	30.8	34	52.3	
**Frequency of sexual intercourse**	2 (wk)-1	5	3.3	57	37.7	89	58.9	0.05=*p
	3 (wk)-2	9	7.9	47	41.2	58	50.9	
	4 (wk)-3	5	12.5	18	45.0	17	42.5	
	> 4 (wk)	1	7.1	6	42.9	7	50.0	
	1–2 (month)	0	0	14	27.5	37	72.5	

*Chi-square test, Significance level: p < 0.05, bold entries are significant results and are related to statistical test

#### Reproductive desire correlates

The desire to reproduce had a statistically significant relationship with the age, the educational level of participants and their spouses, occupation, and socio-economic status. Reproductive intention had a significant inverse relationship with age, whereby the intention to reproduce decreased with increasing age. The desire to reproduce increased alongside the education levels of participants and their spouses. The desire to reproduce in those working was less than in housewives. Participants with a favorable socio-economic level intended to reproduce more so than those with unfavorable and relatively favorable socio-economic levels (Table 1).

## Discussion

Through our findings, we present comparisons between the desire to reproduce and its causes, and lack of desire to reproduce in Iranian cisgender women before and during the COVID-19 pandemic. In the period prior to the pandemic, 51% did not want to have children at all, though this number increased to 57.5% during the pandemic. The intention to reproduce declined from 11.4% in the pre-pandemic period to 5.4% during the pandemic. The most common reason given for not wanting to have children in the pre-pandemic period was not having children already. During the pandemic, this changed to having already reached the desired number of children. The most common reason for not wanting to have children in both periods was having enough children already. Interestingly, 5.5% did not want to have children during the pandemic due to fear of the pandemic itself. The desire to reproduce had a statistically significant relationship with participants’ age, the educational level of participants and their spouses, occupation, and socio-economic status.

The results presented here reflect those reported elsewhere, both in the USA and Italy [14, 16, 18, 19]. Furthermore, in Burkina Faso, a decrease in the desire to have children was reported in comparison to the pre-pandemic period [22]. In Nigeria, 8.8% also reported a lesser desire to reproduce due to concerns about the COVID-19 pandemic [24]. In Tehran, approximately three quarters of participants within a separate study similarly reported no intention to reproduce [21]. Thus, the findings presented here add to the body of emerging evidence in relation to reproduction during the COVID-19 pandemic.

Reproductive intentions can be affected by financial problems, humanitarian crises, an escalation of violence and displacement, or immediate threats such as with the COVID-19 pandemic [25]. Not surprisingly therefore, these have been reported as reasons for a decrease in the desire to have children following the COVID-19 pandemic [13, 14, 19]. Yet in Kenya, participants reported stable desires to reproduce in terms of ideal number of children and timing of pregnancy during the COVID-19 pandemic [26]. Elsewhere, participants considered having a child as a mechanism to deal with social changes caused by the pandemic, with changing working conditions, increasing happiness with having a child, and increased paternal participation in child rearing listed as other causes of an increased desire to reproduce in response to the pandemic [27]. In Italy, the decision to make life changes and the need to be positive were also cited as the main reasons for wanting to reproduce during the pandemic [16]. Indeed, in Iran, the joy of having children is one of the strongest motivations for a couple to have at least one or two children [28]. Future pandemics may also act as a catalyst for similar decision making.

Similar to the findings reported here, in Nigeria, 8.8% of participants changed their minds about getting pregnant due to concerns about the COVID-19 pandemic [24]. In Italy, concerns about future economic problems were also similarly among the main reason for abandoning the intention to reproduce during the pandemic [16]. People have also reported lack of knowledge about gynecological care [27], and the stressfulness of the pandemic as reasons for not wanting to have children [19, 27], and so seemingly, pandemics can act as a catalyst for reproduction or a desire to avoid it.

We found a statistically significant relationship between participants’ age and the desire to reproduce, whereby the desire to reproduce was higher in younger participants. This finding has similarly been reported elsewhere [29–31], and may be due to people approaching the end of their reproductive lives [32], and the fear of health risks [29]. Yet elsewhere in Iran, a positive relationship between advancing age and the intention to reproduce has been observed [21]. This may be due to marriage and the birth of one’s first child occurring at a higher age to begin with [21]. Similarly, in Italy, where the average age at which one births their first child is 31.2 years old [33], the intention to reproduce was found to be higher in those of older age [16]. Such changes may be socially motivated and require further exploration.

In the present study, the desire to reproduce increased with a higher level of education, the main reason for which was the lower number of children in people with a higher level of education and not achieving the desired number of children. These findings are similar to those reported in Australia [34], and the USA [18], and may be associated with having a higher income [35]. Yet in Tehran prior to the COVID-19 pandemic, intention to have children decreased with higher levels of education [36]. This may be because education leads to the formation of more critical thinking, and thus indirectly leads to a decrease in reproduction [37].

Here, the desire to reproduce was shown to increase in those whose spouses had a higher level of education. This finding has also been reported from research conducted in Germany [38], yet not in Torbat Heydarieh (Iran), where no significant relationship was reported [39]. The desire for employed participants to reproduce was less than that of housewives. This may be due to increased independence, rationalism and utilitarianism in working populations [40], and is also the case as reported in Germany [38]. Again, another study conducted in Tehran found no significant relationship in this regard [29]. Conversely, in Australia, the desire to reproduce increased among participants employed during the COVID-19 pandemic [34]. Such increased desires may be due to a decrease in working hours during the pandemic along with socioeconomic and lifestyle changes.

As has been found previously in Iran [36, 39], no statistically significant relationship was found between the desire to reproduce and spouses’ occupation. Also in Iran, spouses’ employment did not have a significant relationship with the intended number of children, while the number of intended children was lower in spouses with higher occupational status [41]. This may be due to negative relationships with the number of intended children and one’s socio-economic status [41]. Nevertheless, such findings both confirm and build upon understandings in this field, particularly in Iran.

Consistent with results reported by Akinyemi et al. (2022), there was no statistically significant relationship identified between participants’ place of residence and their desire to reproduce. Yet the desire to reproduce in Australia during the pandemic was reportedly higher in urban populations than in rural populations [34]. While Abbasi-Shawazi et al. (2022) reported a significant relationship between participants’ place of residence and reproductive intentions, those raised in an urban environment desired fewer children than those in rural areas [29]. In the present study, the desire to reproduce was higher in favorable socioeconomic level than in other levels. This has also been found to be the cases in both Australia and the USA [20, 33] and previously in Iran [42]. Declining rates of reproduction during the pandemic have been reportedly greater in those with a low-income than in those with a higher income [14, 24]. This may be due to a greater vulnerability to economic instability, exacerbated as a result of the COVID-19 pandemic. Elsewhere in Iran, the desire to reproduce increased with income [40]. The reason for these findings may be linked with the lesser value placed on reproduction by those in the upper socio-economic strata of Iranian society [41]. Equally, as income increases, there is typically a preference for fewer, but better educated children [43]. Such findings may usefully inform future population growth strategies.

A key strength of this study is that it included a relatively high sample size and used multi-stage sampling from all 6 districts of Babol city. Yet due to the fact that sensitive questions related to fertility, the possibility of bias in the telephone interview would have been more than in person sampling or participants self-completion of the questionnaire. However, due to restrictions associated with the COVID-19 pandemic, neither in-person sampling nor participants’ self-completion of the questionnaire were possible. Thus recruitment via telephone was unavoidable, and recall bias may have been apparent when answering questions related to the pre-pandemic period.

Considering our findings in relation to the difference in the impact of the desire to get pregnant, future studies may usefully be repeated in other regions to investigate the impact of the COVID-19 pandemic on the desire to be fertile elsewhere. Future research could usefully explore the effects of reduced reproductive desires on birth rates worldwide, and investigate whether such decreases may lead to significant changes in population levels or future birth rates.

## Conclusion and implication of findings

Whilst some may hypothesize that the restrictions and lockdowns associated with the COVID-19 pandemic may have resulted in higher birth rates and increased desires to reproduce, our findings demonstrate the opposite, whereby the COVID-19 pandemic had a negative impact on people’s desire to reproduce. Economic problems caused by the sanctions which increased during the COVID-19 crisis may be one of the reasons for a reduction in people’s intentions to become parents. Considering the effect of the COVID-19 epidemic on the desire to get pregnant, this study can provide a useful basis for conducting further interventional studies to address areas of concern.

## Electronic supplementary material

Below is the link to the electronic supplementary material.

Supplementary Material 1

## Data Availability

All the information obtained from this study is not available to the public due to the confidentiality of the information, but it can be made available upon reasonable request through the Corresponding author.
