# Supplementary material for: Comparing reproductive intentions before and during the COVID-19 pandemic: a cross-sectional study
Source: BMC Health Serv Res. 2023 May 25;23:545. doi: 10.1186/s12913-023-09551-z (PMC10209932; doi:10.1186/s12913-023-09551-z)
Supplement: Supplementary file 1 — Supplementary Material 1 [file 12913_2023_9551_MOESM1_ESM.docx]

**Supplementary file 1. Frequency distribution of allocation of a proportion of selected urban and rural centers of Babol city**

| **Ratio of the number of samples to the total sample (%)** | **Ratio of the number of samples to the population of the center (%)** | **number of samples** | **population covered by center** | **Selected health centers** | **Number of urban/rural health centers** | **Strata** |
| --- | --- | --- | --- | --- | --- | --- |
| 21.5 | 1.27 | 91 | 7212 | **Aliebn Mosareza** | **10 urban health centers** | **Strata 1: central district** |
| 11.6 | 1.26 | 49 | 3875 | **Razia Kola** |  |  |
| 3.8 | 1.23 | 16 | 1300 | **Shahid Zakarian** |  |  |
| 9/2 | 1.24 | 12 | 964 | **Bisheh sar** | **6 rural health centers** |  |
| 5.9 | 1.26 | 25 | 1986 | **Pain Ganj Afruz** |  |  |
| 4.0 | 1.29 | 17 | 1313 | **Siah Kala Mahalleh** |  |  |
| 5.0 | 1.26 | 21 | 1673 | **Dehak** | **8 rural health centers** | **Strata 2: Laleh abad district** |
| 3.1 | 1.35 | 13 | 961 | **Amin Abad** |  |  |
| 8.1 | 1.29 | 37 | 2892 | **Galugah** | **1 urban health center** | **Strata 3:**  **Bandpey-e Sharqi district** |
| 2.6 | 1.25 | 11 | 876 | **Otaq Sara** | **6 rural health centers** |  |
| 8.5 | 1.25 | 36 | 2871 | **Khush Rudpey** | **1 urban health center** | **Strata 4:**  **Bandpey-e Gharbi District** |
| 2.6 | 1.31 | 11 | 863 | **Salehdar Kola** | **2 rural health centers** |  |
| 3.1 | 1.28 | 13 | 1018 | **Darun Kola-ye Sharqi** | **3 rural health centers** | **Strata 5:**  **Babol Kenar District** |
| 3.4 | 1.24 | 18 | 1455 | **Kola Deraz** |  |  |
| 7.1 | 1.29 | 30 | 2329 | **Gatab** | **1urban health center** | **Strata 6: Gatab district** |
| 5.9 | 1.30 | 25 | 1921 | **Darzikola-ye Akhundi** | **3 rural health centers** |  |
| 100 | 1.27 | 425 | 33509 | **6 urban health centers** | **13 urban health centers** | **Total** |
|  |  |  |  | **10 rural health centers** | **28 rural health centers** |  |
